# How do busy hospital circumstances affect mortality and readmission within 60 days: A cohort study of 680 000 acute admissions in Norway

**DOI:** 10.1016/j.healthpol.2022.05.008

**Published:** 2022-05-21

**Authors:** Sara Marie Nilsen, Andreas Asheim, Fredrik Carlsen, Kjartan Sarheim Anthun, Lars Johan Vatten, Stina Aam, Neil M Davies, Johan Håkon Bjørngaard

**Affiliations:** a Center for Health Care Improvement, St. Olav’s hospital, Trondheim University Hospital, Trondheim, Norway; b Norwegian University of Science and Technology, Department of Mathematical Sciences, Trondheim, Norway; c Norwegian University of Science and Technology, Department of Economics, Trondheim, Norway; d Department of Health Research, SINTFF Digital, Trondheim, Norway; e Norwegian University of Science and Technology, Department of Public Health and Nursing, Trondheim, Norway; f Department of Neuromedicine and Movement Science, Faculty of Medicine and Health Science, NTNU-Norwegian University of Science and Technology, Trondheim, Norway; g Department of Geriatric Medicine, Clinic of Medicine, St. Olavs hospital, Trondheim University Hospital, Trondheim, Norway; h Medical Research Council Integrative Epidemiology Unit, University of Bristol, BS8 2BN, United Kingdom; i Population Health Sciences, Bristol Medical School, University of Bristol, Barley House, Oakfield Grove, Bristol, BS8 2BN, United Kingdom; j Nord University, Faculty of Nursing and Health Sciences, Levanger, Norway

## Abstract

**Objective:**

To study mortality and readmissions for older patients admitted during more and less busy hospital circumstances.

**Design:**

Cohort study where we identified patients that were admitted to the same hospital, during the same month and day of the week. We estimated effects of *inflow of acute patients* and the number of *concurrent acute inpatients.* Mortality and readmissions were analysed using stratified Cox-regression.

**Setting:**

All people 80 years and older acutely admitted to Norwegian hospitals between 2008 and 2016.

**Main outcome measures:**

Mortality and readmissions within 60 days from admission.

**Results:**

Among 294 653 patients with 685 197 admissions, mean age was 86 years (standard deviation 5). Overall, 13% died within 60 days. An interquartile range difference in inflow of acute patients was associated with a hazard ratio (HR) of 0.99, 95% confidence interval (95% CI) 0.98 to 1.00). There was little evidence of differences in readmissions, but a 7% higher risk (HR 1.07, 95% CI 1.06 to 1.09) of being discharged outside ordinary daytime working hours.

**Conclusions:**

Older patients admitted during busier circumstances had similar mortality and readmissions to those admitted during less busy periods. Yet, they showed a higher risk of discharge outside daytime working hours. Despite limited effects of busyness on a hospital level, there could still be harmful effects of local situations.

## 1 Introduction

Increasing the utilisation of unused hospital capacity is common as a health policy goal [1], but having insufficient spare capacity could affect care for the older patient during busy periods. While demand for hospital services varies over time, staffing levels and services are less flexible [2,3]. Being admitted during a busy period could affect mortality and readmissions, particularly if hospitals are operating with limited spare capacity. Since hospitals cannot refuse to admit patients with acute conditions, periods with surges in the number of acute patients could result in a shortage of appropriate human resources, medical equipment, and hospital beds [4].

Busy periods could be particularly stressful for older patients, who are often frail [5,6] due to advanced age and high prevalence of reduced physiological reserves resulting in multi-morbidity. These patients are therefore particularly vulnerable to stressors such as being admitted during busy periods [7–9]. None of the studies in a recent systematic review[3] on the association between hospital capacity strain and mortality addressed potential effects on older patients. Further, most of the reviewed studies were aimed at departments and wards that are typically under much stress, such as intensive care units and emergency departments [3]. A large German study from 2015^4^, which studied occupancy at wards within hospitals, suggested increased in-hospital mortality following increased occupancy. However, older patients will often need services from different departments during a hospital stay and could be susceptible to harmful consequences from reduced resources at the hospital level. As pointed out in the systematic review [3], there is marked heterogeneity regarding definitions of capacity strain, hospital settings, and overall study quality in the field. However, there will likely not be one correct measure or method for this, as different research questions will require their own analytical design. Different data may also require different analytical approaches. For instance, busyness at admission would typically be related to capacity in the emergency department or time to treatment facilities [10,11], while busyness during hospital stay could influence availability to treatment resources and clinical personnel [12] as well as the discharge process [13]. Information about factors that are needed to assess a hospital’s capacity is seldomly included in routinely collected data [4]. Data on admissions and discharge are, however, reliable and could be used to identify more and less busy periods.

Studying effects of hospital utilisation on patient safety is challenging because the case mix of patients admitted during busier circumstances is likely to differ from that of patients admitted at quieter periods. Because administrative data include only limited information about patients’ disease severity, adjustment for available information would not be sufficient to provide comparability [2]. Also, patients with more severe illnesses may be more likely to be treated at larger, busier, and more specialised hospitals. In these situations, the association of busy circumstances with patient outcomes may be confounded by the patients’ comorbidities, many of which may be unmeasured.

In the present study, we investigated potential effects of more and less busy circumstances by analysing time periods where a surge in the number of acute patients would likely be out of the hospital’s control [4]. Further, we hypothesized that possible effects of busier periods for the older patients’ prognosis would be strongest in the first days after admission. Hence, we estimated busyness as an average over the first four days of the patients’ hospital stay [12]. While it is not uncommon to analyse within-hospital effects of busyness [4], we propose an adapted and somewhat more fine grained analytical strategy. Analyses were thus done within groups of patients that were admitted to the same hospital, and during the same month and day of the week. For example, a patient admitted on a Monday in November 2016 was only compared to patients admitted at the same hospital on one of the three other Mondays in November 2016. Such analysis designs have recently been used to study high volumes of surgical admissions, time to surgery, and 60-day mortality among hip fracture patients [11]. Since hospitals are likely not able to respond to changes in busyness over short time spans, Mondays in each month may be thought of as each other’s counterfactual. Thus, potential confounding from differences between hospitals, and hospital-specific changes over time, like organisational changes and seasonal effects, or available resources was avoided or minimized. Within the groups, we assumed that a surge of acute patients could be analysed as a natural experiment [14], where we compared outcomes of older patients who were acutely admitted to hospital during more or less busy circumstances.

## 2 Material and methods

### 2.1 Study setting

As described in a recent Health System Review [15], Norway, as the other Nordic countries, has a universal health and social care system. Further, patient copayments amount to about 15% of healthcare spending, primarily from general practitioner contacts, outpatient clinic visits, medications and dental care. Primary care services are provided by the country’s municipalities, of which there were 356 in 2020. Specialized health services, including hospitals, are nationally owned and funded by the parliament through four regional health authorities that own 20 hospital trusts. Hospital trusts encompass separate treatment locations that could be hospitals as well as smaller units. The organization has varied throughout the study period. In this study, admissions to treatment locations providing acute services were the units of analysis.

Norway, being among the world’s wealthier nations, provides effective and high-quality medical care [15]. The number of physicians and nurses per 1000 inhabitant has increased over the last decades, and is generally among the highest in the EU/EEA [15]. The number of hospital beds is among the highest in the Nordic countries [16], but average bed occupancy is above the OECD-average [15]. The Norwegian health care system is generally considered to perform well [17,18]. However, previous Norwegian studies on hip fracture patients showed an effect of hospitals discharge pressure and high volumes of recent surgical admissions on mortality [11,13].

Patients acutely admitted to Norwegian hospitals have mostly been referred by general practitioners, out-of-hours or nursing home physicians, or they were picked up by ambulance [15]. Further, in most cases, a physician at the emergency department assesses the need for hospitalisation. At the hospital ward, a ward physician decides when the patient is ready for discharge and is responsible for reporting the patient’s care needs to the municipalities responsible for primary care. Local authorities then decide what services the patient will be provided after discharge. On January 1^st^, 2012, the government implemented the Norwegian coordination reform [19]. One feature of this reform was the introduction of a daily financial penalty for patients classified as “ready for discharge and in need of follow-up care in the municipality” [20].

### 2.2 Study cohort

We used data from the Norwegian Patient Registry to acquire information about a nationwide cohort of 294 653 patients with 685 197 acute admissions to hospitals from 1 January 2008 to 31 December 2016. Starting out with 925 425 acute admissions among patients 80 years and older, 20 937 admissions from the two first months of the study period were excluded to ensure that we had information about concurrent inpatient situation, and health care use 60 days prior to admission for all patients. We also excluded 219 291 admissions from the study cohort that occurred within 60 days after an earlier admission. This was done to capture the primary admission if a patient had a series of admissions, where subsequent admissions could be a consequence of conditions during the primary admission. These admissions were however used to measure readmissions.

Admissions (episodes of care) were aggregated from ward episodes as defined in Hassani et al. [21]. Admissions thus start when the patient is admitted to a hospital, through within-hospital transfers and between-hospital transfers, to the discharge of the patient. Each patient was tracked by a unique, anonymous identification number throughout the observation period. This also allowed us to connect patient information from different registries. Information on all-cause mortality during 60 days after admission, not limited to in-hospital deaths, was available for every patient from the Norwegian Cause of Death Registry. Information on contacts with regular general practitioners (GPs) was obtained from the Norwegian Health Economics Administration database (Helfo).

From the Norwegian Patient Registry, we used information about time of admission and discharge of all 5 098 059 acute admissions to identify more and less busy hospital circumstances in the time period. All Norwegian hospital trusts are required to submit information about their clinical activity to the national patient registry [22].

### 2.3 Outcomes

The primary outcomes were mortality and readmission within 60 days from admission. Patients were followed for 60 days, as this time span capture a period when mortality was more likely to be affected by hospital circumstances than by other causes [23]. Mortality, not limited to in-hospital deaths, is available in Norwegian register data. Mortality is not susceptible to selection effects since this information is available for all patients, and it clearly represents a patient safety issue.

Busy hospital circumstances could affect discharge practices [1,13, 24-26]. In this study, we used several such outcomes to capture potential adaptations. Secondary outcomes were thus discharge within 4 days, discharge outside ordinary daytime working hours (6 pm until 8 am), financial penalty (at least 1 day of hospital stay as financial penalty patient), and costs of all hospital use within 60 days of the index admission. Busy hospitals may discharge patients early [13]. Hence, we included the outcomes of being discharged within 4 days, which represents the median length of stay in our cohort. Being discharged outside ordinary daytime working hours when staffing is reduced, also in primary health care, could impair the discharge process, causing, e.g., poor information flow, and it is likely a stressful experience for the patient [24–26]. Hospitals could also have a lower threshold for defining patients as “ready for discharge”, moving them to primary care, during busier periods. Health personnel in Norway have described financial penalty patients, who are waiting in the hospital for primary care services, as being prone to stressful hospital stays [1]. Outcomes indicating subsequent health care use could uncover complications and deterioration from poor treatment during busier periods.

Costs were measured by the weighting of the diagnostic related group [27] (DRG-points) where 1 DRG-point was given a value of 5075 €. The mean exchange rate from 2008 to 2016 was 1 €=8.29 Norwegian kroner, and unit price for 1 DRG point was fixed at 42 081 Norwegian kroner in 2016 [28]. Length of hospitalization was measured in hours and minutes from admission to discharge, converted to days.

### 2.4 Measures

Indicators of capacity like human resources, medical equipment and beds are generally not accessible and uniformly measured on a regular basis [4]. In Norway, however, time of admission and discharge are routinely and reliably registered for all hospital admissions.

By using available information about admission and discharge of all acute patients[4], we defined two indicators of more or less busy hospital circumstances. The indicators were averaged over the first four days of the index patient’s hospital stay (e.g., over the admission day, and day 2 to 4 of admission), representing the median length of stay in our cohort. Hence, any selection effects due to early discharge would be avoided. First, the *inflow of acute patients* was calculated as the mean daily number of acutely admitted patients. Second, the number of *concurrent acute inpatients* was calculated as the mean daily number of acute inpatients present at noon. Indicators were based on the first admitting hospital, excluding the index patient.

Because of differing case-mix between hospitals, and since hospitals share responsibilities with other specialized services and primary care, busyness measures were standardized according to local variability. The busyness measure was constructed to study hospitals’ ability to handle variations in inflow and occupancy of acute patients. To present the results for a substantial but typical change in busyness, the busyness measure was standardized such that, at the same hospital within the same month and the same day of the week, it had a mean of zero and a one-unit change corresponded to the local interquartile range (IQR) of variation. To address potential non-linear effects [4], we used restricted cubic spline regression [29], where the busyness indicators were standardised as z-scores within hospital, month and day of the week. Estimating using splines imposes less restrictions on the form of the associations and could possibly detect non-linear effects like tipping points [4] at the tail end of the distribution of busyness.

### 2.5 Statistical analysis

Because comparability between patients present at different times and hospitals may be limited, we identified patients that were admitted at the same hospital, and during the same month and day of the week (public holidays were coded as Sundays). We defined strata according to these groups, and the estimation was only based on within-strata variability. For example, a patient admitted on a Monday in November 2016 was only compared to patients admitted at the same hospital on one of the three other Mondays in November 2016. Thus, potential confounding from differences between hospitals, and hospital-specific changes over time was avoided or minimized [11]. Examples of this are differences in case-mix between days of the week, seasonal differences in communicable diseases, and abrupt changes in hospital organisation or data registration practice.

Since we compared patients admitted at the same hospital during similar time periods [11,30], and since admission of acute patients are largely out of a hospital’s control [4], we could assume that the potential for confounding was minimised. To investigate the plausibility of this assumption, we estimated the associations between the busyness indicators and baseline indicators of an older acute patient’s condition within the strata. As indicators of possible confounding factors, we used available information about age, sex, hospital admissions during the last 60 days, and the number of visits to a general practitioner during the last 60 days.

Mortality, readmissions, and discharges within 4 days, and discharges outside regular daytime working hours (6 pm until 8 am) were analysed as time-to-event using stratified Cox regression (*stcox*, *strata*) with time from admission as the time scale. For analyses of mortality, patients were followed to death up to 60 days after admission or until the end of 2016, whichever occurred first. For readmissions, patients were followed to readmission or death, up to 60 days or until the end of 2016, whichever occurred first. For discharge within 4 days and discharges outside daytime working hours, patients were followed to discharge or death, up to 4 days from admission or until the end of 2016, whichever occurred first. Stratified Cox could be seen as an analogue to the conditional/fixed effect logistic regression estimation and the fixed effect linear estimation [31]. Analysis of variability in financial penalties was done using a fixed effects logistic regression estimator (*xtlogit*, *fe* in Stata). This was done for the period from 2012 to 2016 as the reform that introduced financial penalties was implemented on January 1^st^, 2012. Hospital costs within 60 days were analysed using a fixed effects linear regression estimator (*xtreg*, *fe* in Stata).

Analyses were adjusted for admission hour with dummy variables for each hour. To improve precision of the estimates we also adjusted for age and age squared, sex, any previous acute admissions 60 days before admission and visits to a general practitioner 60 days before admissions.

We performed sensitivity analyses for all outcomes to investigate if the results were different for winter months (January to March) or not, university hospitals or not, surgical patients or not, before and after 2012, daytime admission (8 am to 6 pm) or not, and weekend admission or not.

To assess potential non-linear effects, we performed analyses with restricted cubic splines regression, comparing outcomes with the mean situation as the reference level (z-score of zero). To investigate if the results could be sensitive to the standardization of the exposure chosen in the main analysis, we also performed the spline analysis with the inflow of acute patients and concurrent patients measured as a percentage of the mean situation within the same month and the same day of the week. Splines were constructed using 6 nodes chosen as recommended by Harrell [29], and confidence bands were obtained with the *xblc* package in Stata.

Precision was evaluated with 95% confidence intervals (CI). In all analyses, standard errors were corrected for within-strata correlation using the cluster-option in Stata. The analyses were performed with Stata version 15.1 and R version 1.1.463. Code for the analyses is available online at GitHub https://github.com/rastlaus/busy_hospital_circumstances. [32].

## 3 Results

Among 685 197 acute admissions in 294 653 patients who were 80 years or older, mean age was 86 years (SD 5 years) and 60% were women, see Table 1. Overall, 13 per cent died within 60 days from admission, and patients were admitted for a median of 4 days (interquartile range (IQR) 5 days). New patients and occupancy at noon shows considerable day-to-day and seasonal variation over the study period, as illustrated by three hospitals in additional figure 1. Medium sized hospitals had a mean daily number of 20 acute admissions (SD 7 admissions) over the study period, see additional table 1.

**Fig. 1:**
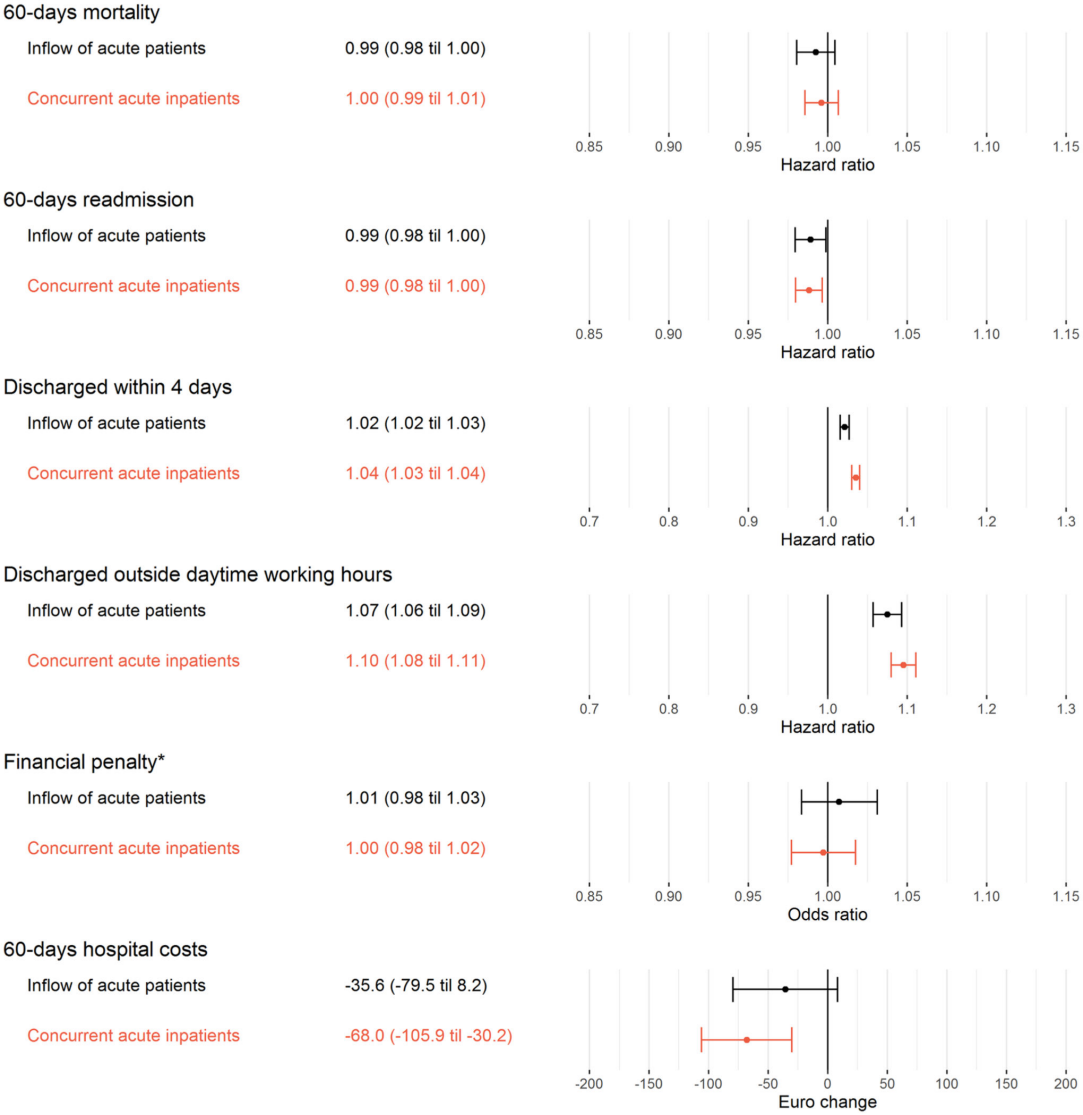
Change in outcomes per interquartile range difference in indicators of hospital busyness. Separate analyses were performed for each outcome. Analyses were done within groups of patients admitted at the same hospital, during the same month, and the same day of the week. All analyses were adjusted for admission hour, sex, age, age squared, GP-visits previous 60 days and acute admission previous 60 days.* Incentive implemented from 1. January 2012. Analyses are thus based on data from 2012 to 2016. Municipalities that postpone follow-up of patients that are defined as ready for discharge and in need of follow up care are charged with a fee per additional day in hospital.

Fig. 1 shows the change in outcomes per interquartile range increase in indicators of hospital busyness. Patients admitted during busier periods had similar mortality to those admitted in less busy periods; a one interquartile increase in inflow of acute patients and the number of concurrent acute inpatients were respectively associated with a hazard ratio (HR) for 60-day mortality of 0.99 (95% confidence interval (95% CI) 0.98 to 1.00) and 1.00 (95% CI 0.99 to 1.01). There was also little evidence for an association with readmission within 60 days of admission (inflow of acute patients: HR 0.99, 95% CI 0.98 to 1.00, number of concurrent acute inpatients: HR 0.99, 95% CI 0.98 to 1.00). However, patients admitted during busier circumstances had a 7% (95% CI 6% to 9%) higher risk of discharge outside daytime working hours. There was little evidence of association of busyness with financial penalties. Patients admitted during busier periods had slightly lower hospital costs within 60 days (-68.0 €, 95% CI -105.9 to -30.2).

**Fig. 2:**
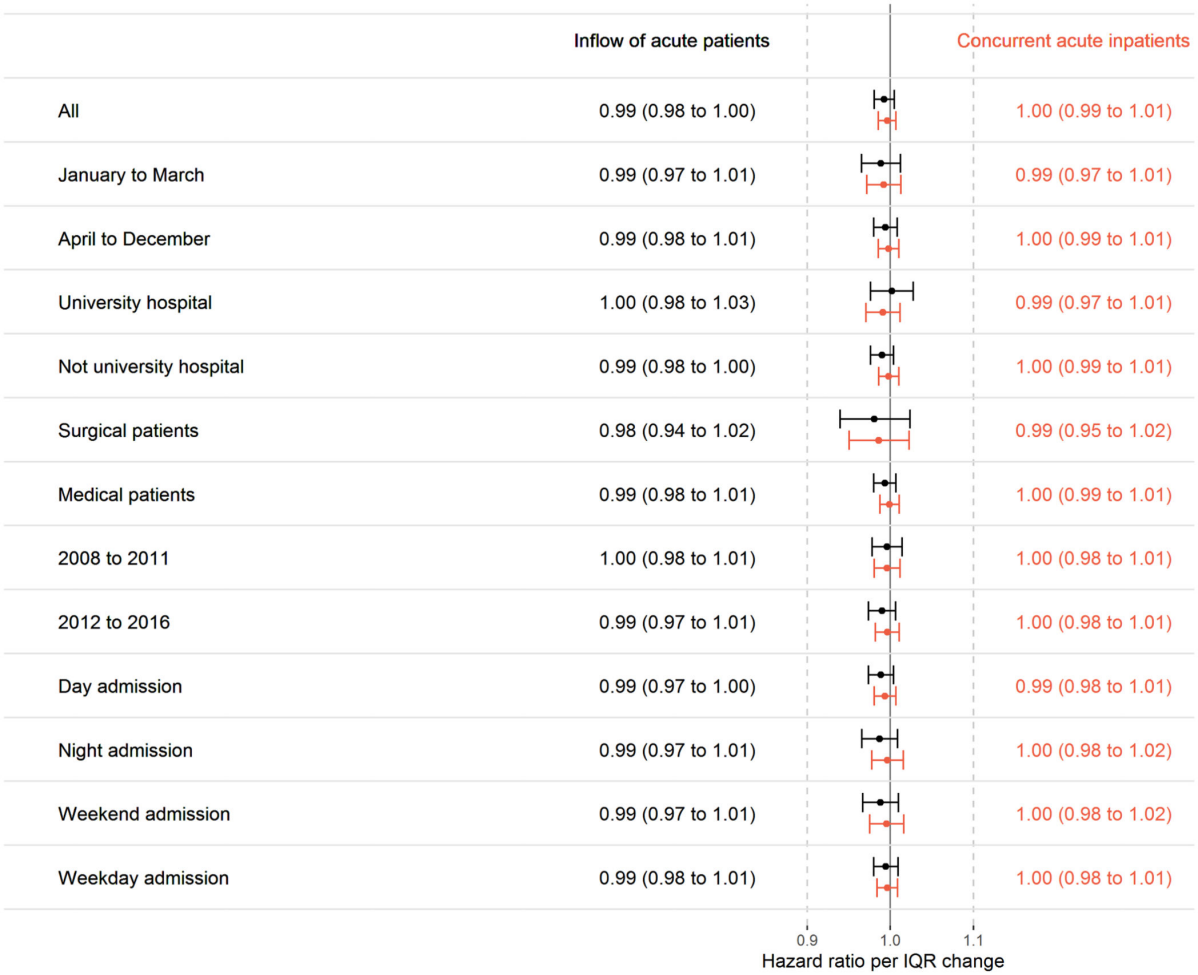
Mortality within 60 days from admission per interquartile increase in indicators of hospital busyness. Separate analyses were performed for each subgroup. Analyses were done within groups of patients admitted at the same hospital, during the same month, and the same day of the week. Also adjusted for admission hour, sex, age, age squared, GP-visits previous 60 days and acute admission previous 60 days.

No subgroups showed any substantial effect of busyness on mortality or readmission 60 days from admission, see Fig. 2 and additional figure 2. Results of subgroup analyses for secondary outcomes (additional figures 3 to 6) were largely the same as in the total material.

**Fig. 3:**
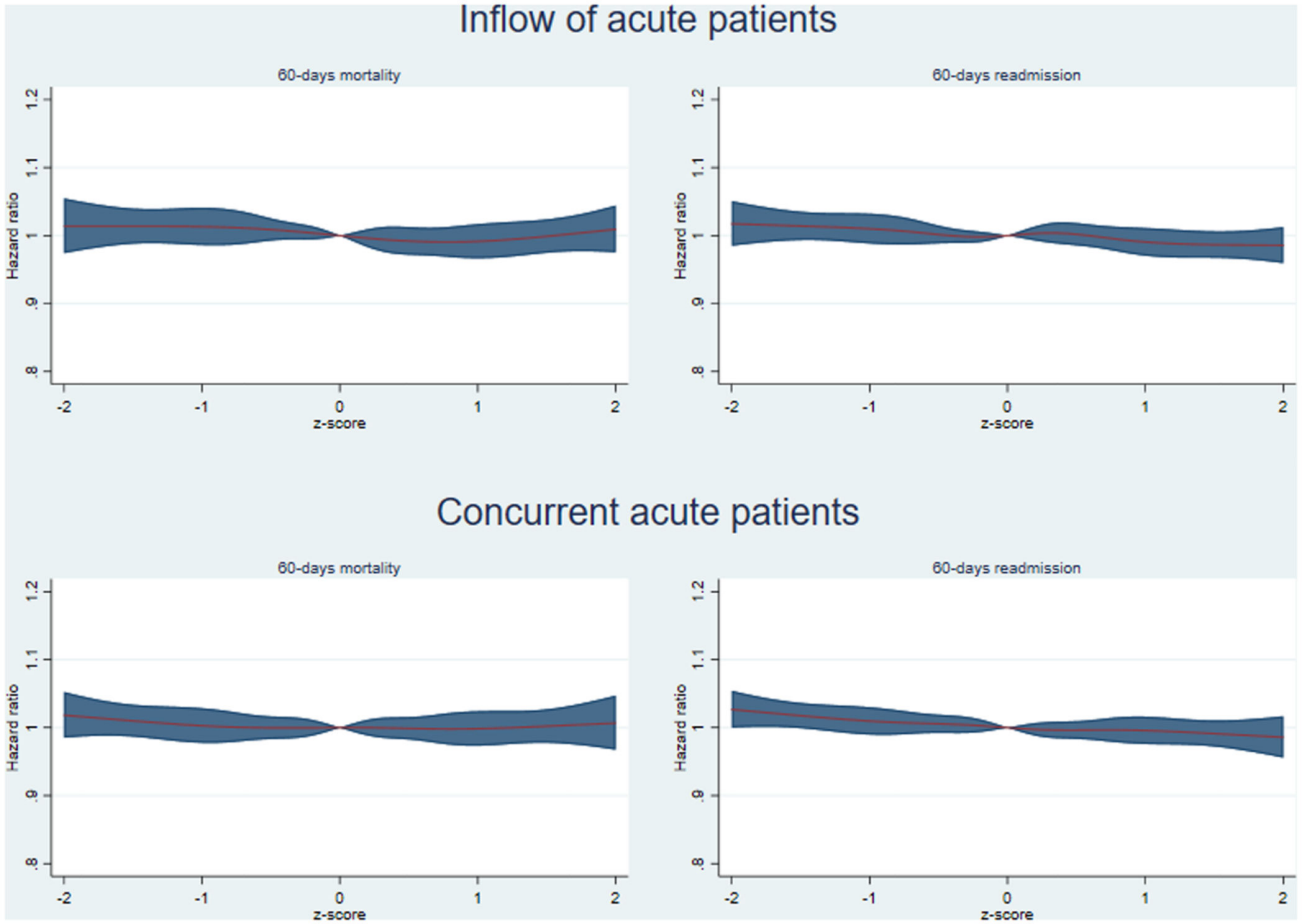
Association between indicators of hospital busyness, 60-days mortality and readmission. Estimated effect measure (red line) with 95% confidence intervals (blue area). Outcomes were compared with the mean situation as the reference level (z-score of zero). Analyses were done within groups of patients admitted at the same hospital, during the same month and day of the week. Also adjusted for admission hour, sex, age, age squared, GP-visits previous 60 days and acute admission previous 60 days.

Spline regression analyses showed no substantial indications of non-linear effects (Fig. 3 and additional figures 7 and 8), nor that the results were sensitive to the choice of exposure standardisation (additional figures 9 to 10).

There was little or no evidence of any differences in potential confounding patient characteristics with busier circumstances within hospital, month and day of the week, see additional table 2.

## 4 Discussion

### 4.1 Principal findings

By using information on admission and discharge for all acute inpatient stays in Norwegian hospitals over a 9-year period, we identified comparable situations where acutely admitted, older patients were exposed to more and less busy hospital circumstances. We found little evidence of a substantial effect on total mortality or readmissions. Yet, the discharge process was somewhat affected, with a higher risk of discharge outside daytime working hours for patients admitted during busier circumstances.

### 4.2 Research implications

Several studies indicate increased mortality for acute patients admitted at weekends relative to weekdays [33,34], and these findings have partly been attributed to confounding by patient-level differences at admission rather than reduced hospital staffing or services during weekends [2]. Studying weekend-effects and potential consequences from being admitted during busier hospital circumstances share some methodological challenges. In the present study, we addressed these concerns by comparing patients who were admitted at the same hospital, month, and day of the week. The patients shared many confounding factors, thus effectively controlling their potential influences. By removing the influence of hospital-specific changes over time, the analyses were not susceptible to confounding due to seasonal phenomena, changes in organization or how data were reported. This approach to analysing routinely collected data from health services may therefore avoid many common pitfalls with such data. Large amounts of data make it difficult to ensure its quality, and biases may easily foil attempts to estimate causal relationships. Due to limitations on data, obtaining a direct measure of a hospital’s capacity for this study was not possible. We approached capacity pressure by studying surges in inflow and occupancy over periods of time where a hospital’s capacity could be assumed largely fixed.

A central assumption in this study was that older patients admitted during busier circumstances should be comparable to patients admitted during less busy circumstances, at the same hospital and during similar time periods. Therefore, we did not rely on adjusting for patient characteristics to ensure comparability. The independence assumption of our busyness exposures was supported by several balance tests on potential confounders. Many studies in the field may have benefitted from a more transparent presentation of the assumptions underlying the estimates.

### 4.3 Health policy implications

In our study, we did not observe any substantial effect on mortality or risk of readmission after being admitted during busier hospital circumstances. The Norwegian health care system is known to provide effective and high-quality medical care [15], and the number of hospital beds is among the highest in the Nordic countries [16]. With this as a backdrop, the service may have handled periods with many acute admissions without affecting mortality and readmissions. However, hospitals may not represent an adequate level of analysis, and our measures might miss out on effects from more local situations. Considering capacity as a hospital or health trust attribute, which often is the case in health policy discussions, may mask potential effects of bottlenecks and more specific challenges. It has, for example, been shown that hip fracture patients admitted to Norwegian hospitals have delayed surgery and higher risk of mortality during situations with many other concurrent acute surgical patients who require immediate care [11]. This issue has also been highlighted in the start of the recent COVID-19 pandemic, where capacity in intensive care units was a challenge, despite a 29 percent drop in acute inpatients in Norwegian hospitals [35].

While we found no effect on mortality and readmission, busier hospital circumstances increased the risk of older patients being discharged outside daytime working hours. Qualitative studies on the hospital discharge process have emphasized that pressure on available hospital beds may have a negative impact on the timing and process of discharge [24–26]. Such concerns are supported, to a certain extent, by our findings. Poorly timed discharges could negatively impact information flow between hospitals and primary health care, since staffing is reduced in evenings and nights, also in primary health care [24]. Further, such discharges are likely a stressful experience, particularly for the older, often frail and multi-morbid patient. Patients in need of home services might be particularly vulnerable for suboptimal co-ordination and collaboration, e.g., in situations when the patient has changed care needs after hospitalisation. Even though we found no effect on mortality and readmission, treatment was to some extent affected by being admitted during busier periods.

### 4.4 Strengths and limitations

This study was based on complete national data from the Norwegian patient registry, which included a large number of older patients. This gave statistical power to detect even very small effects of being admitted during busier circumstances. Our indicators of busyness were based on acute admissions, since these are out of the hospitals control [4]. This reduced the potential for confounding, e.g., from postponing elective treatment of patients during a busier period. To capture effects of busier circumstances during the hospital stay, we measured the indicators of busyness up to day four from admission, which was the median length of stay for the cohort of older patients. This was done without conditioning on discharge, which could introduce selection bias. We excluded admissions that occurred within 60 days after an earlier admission to capture the primary admission if a patient had a series of admissions. This was done as subsequent admissions could be a consequence of conditions during the primary admission. A robustness test, including these admissions, showed similar effects (results not shown). Our analyses were not restricted to in-hospital mortality, a commonly used outcome in studies on hospital capacity strain [3]. This eliminated another potential for bias, since longer hospital stays could give a higher risk of in-hospital death, or other forms of selection bias. Given our model, busier hospital circumstances showed weak or no association with measured patient characteristics, which supports our assumption that patients admitted during busier circumstances were comparable to patients admitted during less busy hospital circumstances, at the same hospital and during similar time periods. Patient experiences and medical errors are examples of other outcomes that could be affected by busier hospital circumstances. Analysing such outcomes were not available from our data and beyond the scope of the present study. Also, busy hospital circumstances may have negative effects on clinical personnel as both sickness absence and work exclusion are known problems among many health care workers [36].

Since this is an observational study, we cannot rule out the possibility that our results could be influenced by residual confounding. However, such residual confounding would only be caused by characteristics that vary within each hospital, month and day of the week. This study was designed to capture effects of being admitted during busier periods, as compared to being admitted during quieter periods. Although all hospitals will experience more and less busy periods, it is a possible weakness of the study that these comparisons would not capture effects of persistent busyness with little variance. Within-group estimators can be susceptible to amplifying the effects from non-differential measurement error [37]. Our independent variables are, however, based on high quality data on admission- and discharge dates, and admitting hospital [22]. The study is based on analysing within groups chosen to minimise confounding, while also retaining variability in hospital busyness. In studies like this, where only variation within groups is analysed, there may be a trade-off between bias and precision.

Since an impact of busyness on patient outcomes would likely be non-linear [4], we performed additional analyses with splines regression. The busyness indicators were here standardised as z-scores and outcomes were compared with the mean situation as the reference level. These analyses showed no indication of an effect on mortality at any level, indicating that our main finding of no substantial effect on mortality and readmission is likely not due to a non-linear effect being masked by an assumption of linearity.

## 5 Conclusions

In this Norwegian cohort study, older patients admitted during busier hospital circumstances had similar risk of mortality and read-mission to those admitted during quieter circumstances. The discharge process was, however, somewhat affected with higher risk of discharge outside regular daytime working hours. Though we found limited effects of busyness on a hospital level, there could still be harmful effects of local situations.

## Figures and Tables

**Table 1:** Descriptive statistics on older patients acutely admitted to hospitals. (N=685 197 admissions)

	N	%
Number of admissions	685 197	
Age in years, mean (SD)	86 (5)	
Women	413 606	60 %
GP-visits previous 60 days	578 439	84 %
Acute admission previous 60 days	41 697	6 %
Non-surgical	579 276	85 %
Mortality within 60 days from admission	90 559	13 %
Readmitted within 60 days from admission	144 581	21 %
Length of hospital stay, median (IQR)	4 (5)	
Discharged outside daytime working hours	110 218	16 %
Financial penalty*	26 206	7 %

SD: Standard deviation. GP: General practitioner. IQR: Interquartile range.

* Incentive implemented from 1. January 2012, N=427 959 admissions from 2012 to 2016. Municipalities that postpone follow-up of patients that are defined as ready for discharge and in need of follow up care are charged with a fee per additional day in hospital.

## Data Availability

The data of this study are publicly available, but restrictions apply to the availability. These data were used under license for the current study.
